# Rivaroxaban versus low-molecular weight heparin plus warfarin prevents portal vein system thrombosis after splenectomy and pericardial devascularization: A randomized clinical trial

**DOI:** 10.17179/excli2020-3120

**Published:** 2021-03-04

**Authors:** Wei Yao, Yongan Feng, Ting Liu, Wujun Li, Mei Zhang, Yingmin Yao, Shengli Wu

**Affiliations:** 1 Department of Hepatobiliary Surgery, the Affiliated Baoji Hospital of Xi'an Medical University, 4 Qingjiang Road, Baoji, Shaanxi 721006, P.R. China; 2 Department of General Surgery, the First Affiliated Hospital of Xi'an Medical University, 48 Fenghao West Road, Xi'an, Shaanxi, 710077, PR China; 3 Department of Hepatobiliary Surgery, the First Affiliated Hospital of Xi'an Jiaotong University, 277 Yanta West Road, Xi'an, Shaanxi 710061, P.R. China

**Keywords:** rivaroxaban, LMWH, warfarin, PVST, splenectomy

## Abstract

This study aimed to evaluate the safety and efficacy of rivaroxaban in preventing portal vein system thrombosis (PVST) in patients with liver cirrhosis after splenectomy and pericardial devascularization. 70 cirrhotic patients undergoing splenectomy and pericardial devascularization were randomly assigned to rivaroxaban treatment (n=35) or low-molecular weight heparin (LMWH) plus warfarin treatment (n=35) for 30 days in this randomized controlled trial. The primary endpoint is the PVST formation. Ultrasound doctors and radiologists were blinded to the randomization results. Both groups received routine outpatient inspection every month and were followed for one year. 17 patients (48.6 %) in rivaroxaban group developed PVST, compared with 27 patients (77.1 %) in LMWH plus warfarin group (*P*=0.025). The incidence of PVST during the first year postoperation was significantly lower in rivaroxaban group than in LMWH plus warfarin group (*F*=7.901, *P*=0.006). The intra-group comparisons versus baseline showed the liver function improved from POD 21 to POM 1, and coagulation function improved from POM 2, in rivaroxaban group. In contrast, the liver function improved from POM 1 to POM 2, and coagulation function improved from POM 4, in LMWH plus warfarin group. The prophylactic use of rivaroxaban significantly decreases the incidence of PVST after splenectomy and pericardial devascularization in cirrhotic patients compared to LMWH plus warfarin treatment. Besides, rivaroxaban treatment was safe and effective and associated with better liver and coagulation functions improvement than LMWH plus warfarin treatment.

## Introduction

Liver cirrhosis is a common chronic progressive liver disease formed by one or more causes, such as hepatitis and chronic alcoholism (Wang et al., 2016[35]). The symptoms of cirrhosis are often absent in early stage, while in the later stage, the main manifestations include impaired liver function and portal hypertension, accompanied by multiple organ function damage (Garcia-Tsao et al., 2017[8]). Currently, surgery is mainly used to treat cirrhosis-induced hypersplenism and prevents upper gastrointestinal hemorrhage, among which splenectomy and pericardial devascularization is one of the most commonly used methods (Chen et al., 2018[3]). Clinical data showed that this operation could effectively prevent the recurrence of upper gastrointestinal bleeding while maintaining hepatic blood perfusion, improving liver function, and improving immunity (Zhao et al., 2013[44]; Ferreira et al., 2007[6]).

A large number of studies have shown that patients with cirrhosis are prone to portal vein system thrombosis (PVST) after splenectomy (Zhang et al., 2016[42]; Jiang et al., 2016[19]). PVST refers to a thrombosis that occurs in portal vein, splenic vein, superior mesenteric vein or intrahepatic portal vein branch, which can lead to the intestinal blood circulation disorder and the decrease of blood flow into the liver (Young and Wong, 2018[40]). If the collateral circulation around the portal vein cannot be established in time, it will lead to the development of intestinal blood stasis, intestinal wall edema and ischemic necrosis, liver ischemia, and even liver failure. All of these will aggravate the portal hypertension and induce upper gastrointestinal hemorrhage and can be potentially fatal (Kline et al., 2019[22]; Hong et al., 2015[13]). Previous research has shown that the incidence of PVST after splenectomy ranged from 8 % to 54 % (Jaime and Pablo, 2017[17]). So far, the detailed mechanisms of PVST formation still remain unclear and have been shown to be related to hemodynamic changes, coagulation mechanism, local vascular pathological changes, as well as perioperative intervention (Wu et al., 2015[37]; Kawanaka et al., 2014[20]).

Up to now, there are few randomized prospective studies on whether preventive anticoagulant therapy should be performed in patients after splenectomy (Bai et al., 2019[2]). Although some studies had shown that the early prophylactic anticoagulation was effective and safe for cirrhosis patients (Zhang et al., 2016[43]; Yang et al., 2015[39]), clinically, many doctors can't decide whether to adopt this controversial therapy at the risk of fatal bleeding, and at present, there is no definite effective preventive anticoagulant regimen.

Low molecular weight heparin (LMWH) is the most commonly used intravenous anticoagulant in clinic (Khorana et al., 2016[21]; Stone et al., 2007[33]), while the most common oral anticoagulation drug is warfarin (Bai et al., 2019[2]). Since the latter takes effect relatively slowly, an overlapping switch from LMWH to warfarin had been adopted for the treatment of PVST (Yang et al., 2016[38]). However, treatment with warfarin has several shortcomings, including easy interaction with other drugs or food, narrow therapeutic window, profound individual difference, and the requirement for frequent dose adjustment (Hirsh et al., 2003[12]; Chua et al., 2015[5]). Rivaroxaban is the first clinically applied direct factor Xa inhibitor, and its efficacy and safety in the prevention of thrombosis in non-valvular atrial fibrillation and orthopedic surgery had been reported by various studies (Shimokawa et al., 2018[32]; Sychev et al., 2019[34]). However, if rivaroxaban could be used for the prevention of PVST is still unknown. 

Therefore, we designed a randomized controlled trial to compare the efficacy and safety of rivaroxaban versus LMWH plus warfarin in cirrhotic patients after splenectomy and pericardial devascularization. The primary endpoint is the PVST formation, and the secondary endpoints include the occurrence of liver decompensation, occurrence of liver cancer, occurrence of active bleeding, withdrawing from the study, and death. 

## Materials and Methods

### Participants

Between January 2017 and May 2019, all cirrhosis patients with secondary hypersplenism admitted at the Department of Hepatobiliary Surgery, the First Affiliated Hospital of Xi'an Jiaotong University were recruited. The inclusion criteria were as follows: Age ≥18 years; Diagnosed with cirrhosis and portal hypertension by clinical evidence, image finding, and histologic examination; Child-Pugh score up to B9; Platelet count ≤50×10^9^/L; With a history of upper gastrointestinal bleeding; No PVST in imaging examinations; No serious heart, lung, kidney and coagulation dysfunction; With written informed consent to join the study. 

The exclusion criteria were as follows: Age >80 years; Combined with liver cancer or other malignant tumors; Combined with other serious organic/infectious diseases, including serious heart, lung, kidney dysfunction and blood system disease, active ulcer hemorrhage, human immunodeficiency virus (HIV) infection; Pregnancy; Ongoing drug treatment, including oral contraceptives, antidepressant/antipsychotic drugs, and anticoagulants; Baseline international normalized ratio (INR) >2; With PVST detected by color Doppler ultrasound or MRI before operation; Declined to participate in this study.

All patients received open splenectomy and pericardial devascularization. All operations were performed using the same procedure as described previously (Chen et al., 2018[3]) by the same group of surgeons. This study was conducted in accordance with the guidelines of the Declaration of Helsinki and was approved by the Ethics Committee of Xi'an Jiaotong University Health Science Centre.

### Study design

The experimental group in our study received oral administration of rivaroxaban (Baoji Guokang Bio-Technology Co., Ltd., Baoji, China) for 30 days beginning on postoperative day (POD) 1 at a dose 10 mg/day. The control group received LMWH (Sanofi-Aventis Pharmaceutical Co., Ltd., Beijing, China) subcutaneously (5000 IU) twice daily beginning on POD 1 for 5 days and oral warfarin sodium (Shanghai HuicH Biotech Co., Ltd., Shanghai, China) beginning on POD 1 for 30 days. The dose of warfarin was 2.5 mg/day initially and then adjusted to reach a target INR of 2-3. Platelet count was routinely monitored in both groups after operation. If the platelet count was > 500 × 10^9^ / L, aspirin (Shanghai HuicH Biotech Co., Ltd., Shanghai, China) was orally given at 100 mg/day for 1 month. If the platelet count was > 1000 ×10^9^ / L, therapeutic plateletpheresis was performed. All patients were followed for 1 year. 

Participants were randomized to experimental group versus control group based on room number (experimental group even; control group odd). Room assignments were done by an independent statistician without knowledge of research details. Patients then received rivaroxaban or LMWH plus warfarin treatment according to different groups. Ultrasound doctors and radiologists were blinded to the randomization results. 

### Efficacy assessment

The primary endpoint of the study is the PVST formation, defined as thrombosis in portal vein, splenic and superior mesenteric veins or intrahepatic portal vein branches. Portal vein system ultrasonography was performed before operation, at 1, 2, 3 and 4 weeks after operation, and every month thereafter. If there was a suspected thrombosis on ultrasound evaluation, abdominal CT would be performed to make an assessment. Secondary endpoints include: (1) occurrence of liver decompensation, including jaundice, ascites, hepatic encephalopathy, spontaneous bacterial peritonitis, variceal bleeding, coagulopathy and hepatorenal syndrome (China et al., 2018[4]); (2) occurrence of liver cancer; (3) occurrence of active bleeding, including the color of drainage became darker or the amount of drainage increased, or the hemoglobin decreased more than 1 g/d; (4) withdrawing from the study, including dropping out of the study due to side effects of drugs or subjective rejection; (5) death from any cause.

Both groups received routine outpatient inspection every month, unless their conditions required other clinical interventions. All patients were followed for one year, except for death or dropping out of the study. We reported this randomized controlled trial according to the most recent CONSORT statement criteria (Schulz et al., 2010[31]).

### Safety assessment

All side effects were classified and recorded in accordance with the World Health Organization toxicity grading system (Franklin et al., 1994[7]). During the course of the study, if the patient has obvious abnormal laboratory test indexes or related adverse events, the drug treatment will be temporarily interrupted. When the above abnormalities or adverse events are solved, the drug use will be resumed; otherwise, the treatment will be terminated.

### Statistical analysis

Data were presented as mean ± standard deviation (SD) and percentage (%) for continuous variables and categorical variables, respectively. Statistical differences between groups were determined by a two-tailed Student's t-test or chi-squared test, using SPSS 18.0 software (SPSS Inc., Chicago, IL, USA). A *P* value <0.05 was considered statistically significant.

## Results

### Trial profile

Overall, 106 cirrhosis patients who received splenectomy and pericardial devascularization were screened for study eligibility. Among them, 34 were excluded due to the combination of HCC (n=7) and malignant tumor of other organs (cervical carcinoma, n=1; lung cancer, n=1), chronic renal failure (n=2), ongoing drug treatment (antipsychotic drugs, n=3; anticoagulants, n=5), existence of PVST (n=11), and refusal to join the study (n=4). Thus, 72 patients were randomly assigned to the rivaroxaban group or LMWH plus warfarin group (n=36 in each group). On POD 3, one patient in LMWH plus warfarin group withdrew from the study due to active bleeding, and another patient in rivaroxaban group stopped taking drugs on her own after leaving the hospital on POD 8. Finally, 70 patients completed the treatment (n=35 in each group).

### Demographic features and baseline characteristics of the patients 

There were no significant differences in demographics, preoperative laboratory indexes, and intraoperative clinical features between rivaroxaban group and LMWH plus warfarin group (all *P* > 0.05; Table 1(Tab. 1)). 

### Dose of warfarin sodium and platelet level

The median dosage of warfarin sodium was 3.5 mg for maintaining INR between 2 and 3 and the dose range was 2.0-7.5 mg during the whole treatment process. The daily dose of warfarin was 3.24 ± 0.25 mg at POD 7, 3.48 ± 0.28 mg at POD 14, 3.87 ± 0.23 mg at POD 21, and 3.87 ± 0.23 mg at POD 30.

There were no significant differences in platelet counts between rivaroxaban group and LMWH plus warfarin group at various time points (all *P* > 0.05; Table 2(Tab. 2)). Two patients in each group accepted aspirin orally for one month due to high platelet count (> 500 × 10^9^/L). Two patients in LMWH plus warfarin group and one in rivaroxaban group received therapeutic plateletpheresis when platelet count was > 1000 ×10^9^/L. There were no significant differences in the proportion of patients receiving aspirin treatment or therapeutic plateletpheresis between the two groups (all *P* > 0.05). 

### Incidence and distribution of PVST after operation

The overall incidence of PVST was 62.9 % (44/70). A total of 17 patients (48.6 %) in rivaroxaban group developed PVST, compared with 27 patients (77.1 %) in LMWH plus warfarin group (*P*=0.025). The incidence of PVST during the first year postoperation was significantly lower in rivaroxaban group than in LMWH plus warfarin group (*F*=7.901, *P*=0.006; Table 3(Tab. 3)). 

There were no significant differences between rivaroxaban group and LMWH plus warfarin group in PVST incidence on POD 7, POD 14, POD 21 and POM (postoperative month) 1 (all *P* > 0.05; Table 3(Tab. 3)). However, from POM 2 to POM 7, the PVST incidence was significantly lower in rivaroxaban group than in LMWH plus warfarin group (all *P* < 0.05; Table 3(Tab. 3)). After that (from POM 8 to POM 12), there were no significant differences in PVST incidence between the two groups (all *P *> 0.05; Table 3(Tab. 3)). 

Of the 17 cases of PVST in rivaroxaban group, there were 4 in the portal vein, 3 in the splenic vein, 1 in the superior mesenteric vein, 5 in the portal and splenic veins, 3 in the splenic and superior mesenteric veins, and 1 in the portal, splenic and superior mesenteric veins. Of the 27 cases of PVST in LMWH plus warfarin group, there were 6 in the portal vein, 5 in the splenic vein, 2 in the superior mesenteric vein, 6 in the portal and splenic veins, 5 in the splenic and superior mesenteric veins, and 3 in the portal, splenic and superior mesenteric veins. It is notable that splenic vein thrombosis occurred in 31 (70.5 %) cases. 

Of the 44 cases of PVST patients, 9 (20.5 %) were symptomatic. 4 of them were from rivaroxaban group and 5 of them from LMWH plus warfarin group (23.5 % *vs.* 18.5 %; *P*=0.716). The symptoms manifested as loss of appetite (2 patients; 1 in rivaroxaban group and 1 in LMWH plus warfarin group), abdominal pain (2 patients; 1 in rivaroxaban group and 1 in LMWH plus warfarin group), fever (4 patients; 1 in rivaroxaban group and 3 in LMWH plus warfarin group), and abdominal distension (1 patient in rivaroxaban group). These symptoms appeared alone or in combination, and all resolved over time.

### Occurrence of liver decompensation

During the first year postoperation, the occurrence of liver decompensation was similar between the two groups (*P* > 0.05; Table 4(Tab. 4)). There were no significant differences between the two groups in the incidence of jaundice, ascites, hepatic encephalopathy, variceal bleeding, and coagulopathy (all *P* > 0.05; Table 4(Tab. 4)). No spontaneous bacterial peritonitis and hepatorenal syndrome occurred in either group.

### Effect on liver function and liver cancer occurrence

Intra-group comparison of liver function [ALT (U/L), total bilirubin (TBIL), albumin (ALB)] at baseline versus at POD 7, POD 14, POD 21, and POM 1 to 12 in rivaroxaban group showed ALT level returned to normal from POD 21, and obvious improvement in TBIL as well as ALB from POM 1 (all* P* < 0.05; Table 5(Tab. 5)). For LMWH plus warfarin group, the intra-group comparison showed ALT level returned to normal from POM 1, and significant improvement in TBIL as well as ALB from POM 2 (all* P* < 0.05; Table 5(Tab. 5)). There were no significant differences in the incidence of hepatocellular carcinoma between the two groups (two in each group; *P*=1.000). 

### Effect on INR and active bleeding

Intra-group comparison of INR at POD 7, POD 14, POD 21, and POM 1 to 12 versus at baseline in rivaroxaban group showed a slightly but not significantly increased INR from POD 7 to POM 1 (all *P* > 0.05; Table 5(Tab. 5)), and a significantly decreased INR from POM 2 to POM 12 (all *P* < 0.001; Table 5(Tab. 5)). For LMWH plus warfarin group, the intra-group comparison showed a significantly increased INR from POD 7 to POM 1 (all *P* < 0.001; Table 5(Tab. 5)), a similar INR to baseline from POD 2 to POM 3 (Table 5(Tab. 5)), and an obviously decreased INR from POM 4 to POM 12 (all *P* < 0.001; Table 5(Tab. 5)).

There were no significant differences in the incidence of active bleeding (one patient in LMWH plus warfarin group withdrew from the study due to active bleeding; *P*=1.000).

### Safety and survival

All patients could tolerate rivaroxaban well. In warfarin group, one patient stopped treatment because of active bleeding on POD 3. Three patients stopped warfarin treatment for 1 to 2 days due to abnormally high INR value (> 3). One patient had warfarin resistance. Although the dose of warfarin was increased to 7.5 mg/day, the INR value of the patient continued to be lower than 2 and therefore warfarin was administered at 7.5 mg/day until the end of the 1^st^ month treatment. All patients were in good condition when they left hospital. No patient died during the 1-year follow-up period.

See also the Supplementary data.

## Discussion

PVST is one of the common complications after splenectomy. It may lead to the decrease of portal vein blood flow, aggravate the damage of liver function, increase the portal vein pressure as well as the risk of gastrointestinal rebleeding, and even threaten the life of patients. However, there is no standard protocol for the prevention of PVST in patients after splenectomy and pericardial devascularization to date, and an effective and safe anticoagulant regimen is thus urgently needed. 

Previous studies have shown that the formation of postoperative PVST is mainly related to the patient's own factors, hemorheology changes, coagulation mechanism and perioperative treatment intervention (Hirofumi et al., 2014[11]; Gelas et al., 2014[9]). The elevated portal vein pressure in patients with cirrhosis and portal hypertension could lead to the increase of the collagen fibers and extracellular matrix in vascular wall, and the adherence of platelets to injured endothelial cell would lead to thrombosis occurrence (Jiang et al., 2016[18]). In addition, the size of spleen of patients is closely related to the PVST formation. The larger the spleen is, the greater the impact of splenectomy on portal hemodynamics is. Previous study showed that thickness of spleen, splenic vein diameter and portal vein diameter were all risk factors of PVST formation after splenectomy in patients with liver cirrhosis (Wu et al., 2015[37]; Qian and Li, 2017[29]).

LMWH is one of the most commonly used anticoagulant drugs. A previous study reported that LMWH (Enoxaparin) could effectively and safely prevent PVST and liver decompensation in patients with advanced cirrhosis (Yuko et al., 2018[41]). However, in that study, patients need to receive LMWH subcutaneous injection 336 times, which not only means a very high compliance requirement, but also a high treatment cost for patients. Besides, LMWH is not suitable for patients with bleeding tendency or active bleeding because it increases the risk of bleeding and causes heparin induced thrombocytopenia (Pollak, 2019[28]). In contrast, warfarin is cheap and requires only low-level compliance, and overlapping treatment with LMWH and warfarin had been reported in a few studies. Lai et al. (2012[24]) reported that compared with control group (received aspirin or warfarin without LMWH irregularly), patients treated with LMWH for 5 days, followed by oral warfarin and aspirin for one month had a significantly decreased PVST (20.94 % *vs. *41.17 %). However, this is a retrospective study and the actual PVST incidence might be higher than they reported. Ikeda et al. (2005[16]) reported that 67 % of PVST cases were asymptomatic and diagnosed after a contrast enhanced CT scan screening. Since most patients were asymptomatic, the real PVST incidence is probably underestimated. Another study by Sanchez-Ocaña et al. (2019[30]) reported that initial anticoagulation with LMWH started on day 4 or 5 post-transplant for 4 weeks followed by oral warfarin for six months could effectively prevent the reoccurrence of PVST. However, this is also a retrospective study and its focus is to prevent recurrence rather than occurrence of PVST.

Recent studies had reported the use of rivaroxaban in the treatment of PVST (Ai et al., 2020[1]; Pannach et al., 2013[27]; Naymagon et al., 2020[25]). However, the preventive effect of rivaroxaban on PVST has not been reported. In this 1-year randomized controlled trial (RCT) in cirrhotic patients undergoing splenectomy and pericardial devascularization, the efficacy and safety of prophylactic application of rivaroxaban had been demonstrated. Treatment with rivaroxaban significantly reduced the incidence of postoperative PVST formation, alleviated liver injury, improved coagulation function, compared with LMWH plus warfarin treatment. As far as we know, this is the first RCT supporting the preventive effect of rivaroxaban on PVST.

In our study, we found that the incidence of PVST in rivaroxaban group was lower than that in LMWH plus warfarin group at all time points from POD 7 to POM 12, and the difference between the two groups was significant from POM 2 to POM 7 (all *P* < 0.05). During the first year postoperation the incidence of PVST in rivaroxaban group was significantly lower than that in LMWH plus warfarin group (*F*=7.901, *P*=0.006). Among the total 44 PVST patients, splenic vein thrombosis appeared in 70.5 % of cases. This result is consistent with previous reports that hemodynamic changes of the portal venous system played an important role in the formation of PVST (Wei et al., 2017[36]; Huang et al., 2018[15]). The blood turbulence or congestion in the splenic vein stump leads to the deposition of blood cell components, which eventually leads to splenic vein thrombosis, and then it can extend to the portal vein and superior mesenteric vein (Wu et al., 2015[37]). 

The liver function (ALT, TBIL, ALB) and coagulation function (INR) in both groups gradually improved during the first year postoperation. The intra-group comparisons versus baseline showed the liver function improved from POD 21 to POM 1 in rivaroxaban group. In contrast, these parameters were ameliorated from POM 1 to POM 2 in LMWH plus warfarin group. For coagulation function, the intra-group comparisons showed the INR decreased versus baseline from POM 2 in rivaroxaban group. In contrast, a decreased INR could not be observed until POM 4 in LMWH plus warfarin group. The difference in liver function recovery might be related to the lower PVST incidence in rivaroxaban group, since the PVST could decrease blood flow into the liver and lead to liver damage. As for the difference in coagulation function recovery, it could be attributed to the different mechanisms of action and drug properties of anticoagulants. Warfarin, a dicoumarin derivative, acts as an anticoagulant by inhibiting the synthesis of vitamin K-dependent coagulation factors II, VII, IX and X in the liver. However, its safe use is restricted by a narrow therapeutic window and large individual differences (Guo et al., 2006[10]) and therefore must be used with INR monitoring to keep the INR value between 2 to 3. Rivaroxaban is a new oral anticoagulant, which can selectively inhibit the active site of factor Xa and block internal and external coagulation pathways simultaneously (Kvasnicka et al., 2017[23]). Compared with warfarin, rivaroxaban has the advantages of high bioavailability and no need for INR monitoring, although a previous study demonstrated that INR was significantly elevated in 84.2 % of the patients treated with rivaroxaban with a median INR value of 1.7 (Ofek et al., 2017[26]). Our result is consistent with this report that the INR was slightly but not significantly increased in patients during rivaroxaban treatment. Thus, it is reasonable that the INR value in rivaroxaban group returned to normal more quickly than in LMWH plus warfarin group. 

Although some studies (Bai et al., 2019[2]; Pollak, 2019[28]; Hongwei et al., 2015[14]) have suggested that postoperative application of LMWH or/and warfarin in cirrhotic patients with portal hypertension after splenectomy was safe, the prophylactic use of anticoagulants is still limited clinically due to the concerns about the increased risk of bleeding. Our study affirmed the results of previous studies showing the safety of LMWH plus warfarin for these patients. More importantly, we provided the first evidence that rivaroxaban at a dose of 10 mg/day for 1 month was safe and more effective than LMWH plus warfarin for the prevention of PVST after splenectomy and pericardial devascularization. Besides, the liver and coagulation functions recovered more quickly in patients treated with rivaroxaban than in patients treated with LMWH plus warfarin. 

There are potential limitations of the current study. First, the sample size is relatively small. Second, this is a single-center study and the results may therefore be affected by local conditions. Our study included a cohort of Chinese cirrhosis patients with secondary hypersplenism arising from mainly hepatitis B, who accepted splenectomy and pericardial devascularization. Third, a longer follow-up time is required to validate our findings.

In conclusion, this 1-year RCT demonstrated that the prophylactic use of rivaroxaban from POD 1 at a dose 10 mg/day for 30 days was safe and effective for the prevention of PVST after splenectomy and pericardial devascularization in cirrhotic patients. In addition, treatment with rivaroxaban was associated with (better) improvement in liver and coagulation functions than treatment with LMWH plus warfarin.

## Notes

Wei Yao and Yongan Feng contributed equally as first authors.

Yingmin Yao and Shengli Wu (Department of Hepatobiliary Surgery, the First Affiliated Hospital of Xi’an Jiaotong University, 277 Yanta West Road, Xi’an, Shaanxi 710061, P.R. China; victorywu2000@163.com) contributed equally as corresponding authors.

## Conflict of interest

The authors declare no conflict of interest.

## Ethics approval

This study was conducted in accordance with the guidelines of the Declaration of Helsinki and was approved by the Ethics Committee of Xi’an Jiaotong University Health Science Centre.

## Funding

This study was supported by grants from the Key Research and Development Program of Shaanxi Province [no. 2017SF-195 and no. 2020SF-060] and Natural Science Fund for Science and Technology Innovation in Ali Region of Tibet Autonomous Region [no. akkczrjj20180207].

## Authors' contributions

Concept – Yingmin Yao, Shengli Wu

Design - Shengli Wu

Supervision - Shengli Wu

Funding - Wujun Li, Shengli Wu

Data Collection and/or Processing - Wei Yao, Yongan Feng, Ting Liu, Wujun Li

Analysis and/or Interpretation - Mei Zhang, Shengli Wu

Literature Review and Writer - Shengli Wu.

## Trial registry number

We registered our research at https://www.clinicaltrials.gov/. The name of research registered is “Preventive effect of rivaroxaban on portal vein thrombosis after splenectomy in cirrhotic patients with portal hypertension”. The trial registration identifier at clinicaltrials.gov is NCT04397289.

## Supplementary Material

Supplementary data

## Figures and Tables

**Table 1:**
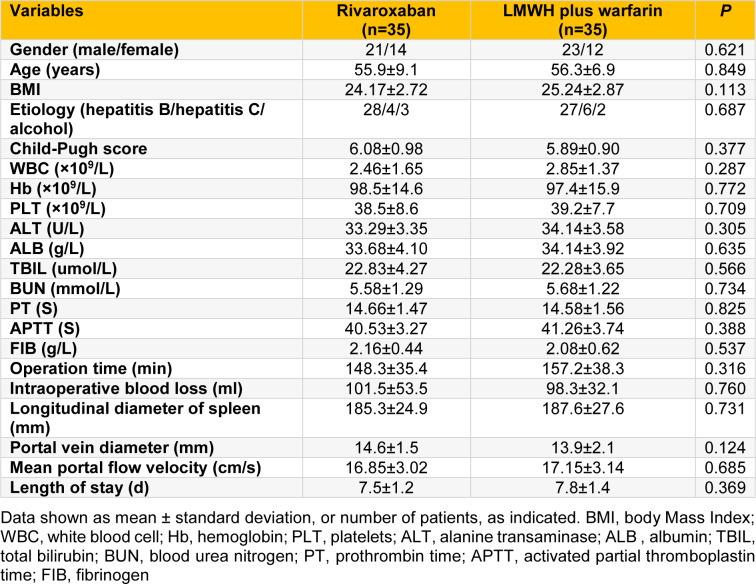
The demographics, preoperative laboratory indexes, and intraoperative clinical features of the two groups

**Table 2:**
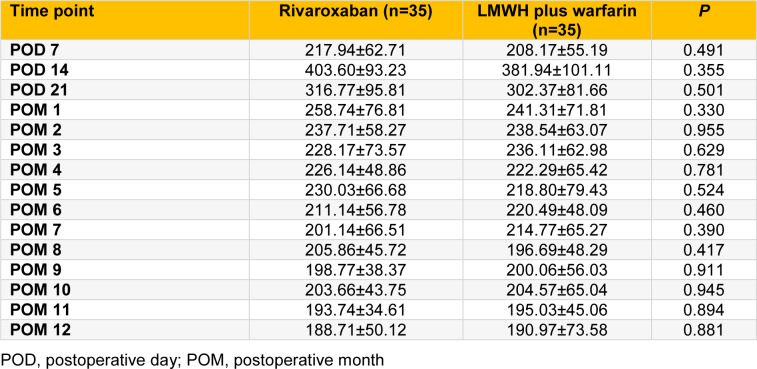
The comparison of platelet level between rivaroxaban and LMWH plus warfarin groups

**Table 3:**
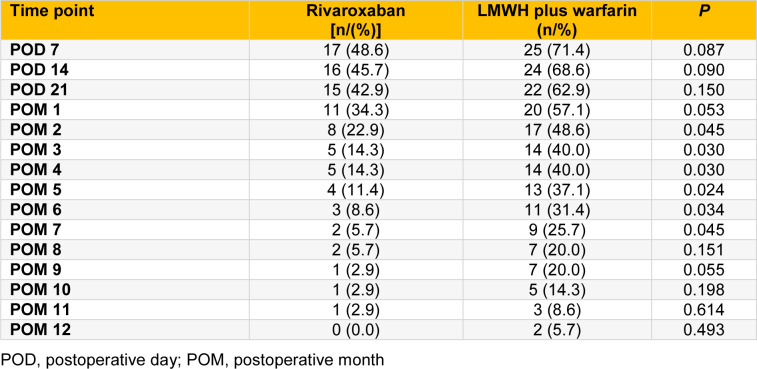
The comparison of PVST incidence between rivaroxaban and LMWH plus warfarin groups

**Table 4:**
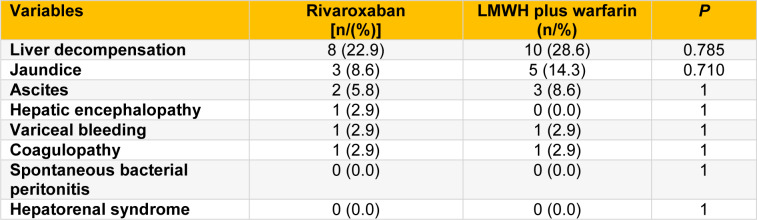
The incidence of liver decompensation in rivaroxaban and LMWH plus warfarin groups during the first year postoperation

**Table 5:**
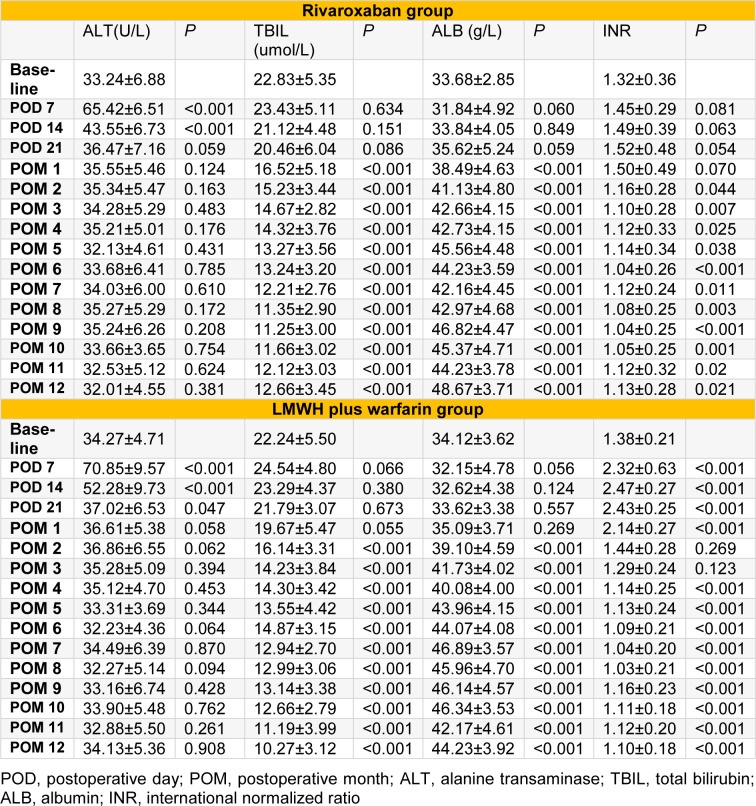
Markers of liver function (ALT, TBIL, ALB) and coagulation function (INR) in rivaroxaban and LMWH plus warfarin groups
